# Effects of chromium propionate supplementation on production performance, blood parameters, ruminal fermentation indices, and microbial diversity in heat-stressed Holstein dairy cows

**DOI:** 10.3389/fvets.2025.1651670

**Published:** 2025-09-01

**Authors:** Bihong Zhang, Yongqiang Wen, Zixin Zhang, Qimin Liu, Yazhou Wang, Chenxu Zhao, Jianguo Wang

**Affiliations:** College of Veterinary Medicine, Northwest A&F University, Yangling, China

**Keywords:** heat stress, dairy cow, chromium propionate, production performance, physiological function

## Abstract

Global warming increases the risk of heat stress in dairy cattle, thereby jeopardizing their health and the economic benefits of dairy farms. Chromium propionate (CrPro) is a chromium source permitted for use in feed additives. However, research on the effects of CrPro on heat-stressed dairy cows is limited. Therefore, this study assessed the effects of different doses of CrPro on heat-stressed dairy cows. Holstein dairy cows with similar body condition, milk yield, and parity were randomly divided into three groups: the control group (Con), the low-dose group (CrL), and the high-dose group (CrH), with 10 cows per group. CrPro was supplemented at doses of 0, 4, and 8 mg/(d·cow) in the basal diets of each group, respectively. The trial lasted for 45 days. The temperature-humidity index (THI) in the cowshed was monitored daily to assess the heat stress status of the cows. Daily milk yield was recorded, and rectal temperature was measured according to experimental requirements. Milk composition, antioxidant capacity, liver enzyme activity, lipid metabolism, and other parameters were analyzed. The composition and diversity of the rumen microbiota were also assessed. Results showed that the average THI in the cowshed during the trial period (0–45 days) was 74.97 ± 2.85, indicating that dairy cows used in this study experienced mild heat stress. Compared with the Con group, the CrL and CrH groups had reduced rectal temperature and respiratory rate. The CrH group had significantly lower milk yield loss and somatic cell count (*p* < 0.05). No significant differences were observed in milk composition, antioxidant capacity, liver enzyme activity, or lipid metabolism between the CrL and CrH groups (*p* > 0.05). Compared with the CrH group, the CrL group had lower volatile fatty acids (VFAs) (acetate, propionate, butyrate, isobutyrate, valerate, and isovalerate) in rumen fluid. Compared with the control group, both CrL and CrH groups exhibited enhanced microbial abundance and an optimized ruminal community structure. Overall, an 8 mg/(d·cow) dose of CrPro had a greater impact on improving production performance and economic benefits. The findings of this study provide data support for selecting an appropriate CrPro dosage to reduce heat stress-induced economic losses on dairy farms.

## 1 Introduction

Temperature is a critical factor influencing livestock production, closely related to the health, regular reproduction, and milk quality of dairy cows (1). Climate change has led to more frequent extreme weather conditions, causing increased temperatures and reduced livestock productivity, resulting in economic losses (2). Dairy cows are highly sensitive to heat stress, exhibiting abnormal changes in body temperature regulation, production performance, reproductive performance, and immune function (3). Naturally, cows accumulate a significant internal heat load due to metabolism for milk synthesis (4). Excessive exposure to high temperatures and humidity can weaken their capacity to dissipate accumulated heat, typically quantified by the temperature-humidity index (THI) (4). THI objectively reflects the degree of heat stress in the environment and is widely used and validated on most farms. The formula for THI is: THI = 0.81 T + (0.99 T − 14.3) RH + 46.3, where T and RH represent temperature and relative humidity, respectively (5). A THI of 72–79 indicates mild heat stress, 79–88 denotes moderate heat stress, and values >88 signify severe heat stress. Rectal temperature (RT) and respiratory rate (RR) provide direct indices for assessing the intensity of heat stress in animals (6). RT exceeding 38.5°C usually indicates heat stress. The normal RR for dairy cows is 26–50 breaths per minute. During heat stress, cows attempt to dissipate excess body heat by increasing RR, which can exceed 80 breaths per minute in severe cases (7). O’Brien et al. (8) found that as THI increases, calf feeding time significantly decreases, and dry matter intake (DMI) drops by about 12%. Heat-stressed cows require substantial water supplementation, with an increase of 1.2 kg in water intake per cow for every 1°C rise in ambient temperature. Research indicates that for each unit increase in THI above 70, daily milk yield declines, feed intake decreases, and milk composition is adversely affected (9). Higher THI values lead to a decline in milk fat and protein percentages, reducing milk quality (9). Concurrently, heat stress perturbs blood components, cellular energy metabolism, the immune system, oxidative stress, and hormone levels in cows (10, 11). It also disrupts impacts normal rumen metabolism in cows (12). Zhao et al. (13) demonstrated that heat stress increases the relative abundance of lactate-producing bacteria while decreasing the relative abundance of acetate-producing bacteria. Tajima et al. (14) also demonstrated that heat stress significantly affects the rumen microbial composition of Holstein heifers.

Chromium is present in trace amounts in forage and grains (15). Typically, chromium supplements (Cr^3+^) are added to total mixed rations to meet the nutritional needs of dairy cows (16). Chromium supplementation is involved in carbohydrate metabolism in animals, improving glucose tolerance (17). Chromium can stimulate insulin-dependent glucose metabolism in fat cells and activate phosphotyrosine phosphatase on fat cell membranes (18, 19). Chromium supplementation can also alleviate liver damage in a rat model of chronic cholestasis (20). Research has found that chromium supplementation not only enhances the immune response, feed intake, and milk production in dairy cows but also increases the blood enzyme activities of glutathione peroxidase (GSH-Px) and superoxide dismutase (SOD) (21). Moreover, chromium yeast modulates the rumen microbiota and amino acid metabolites in heat-stressed dairy cows to promote milk protein synthesis (12). The Center for Veterinary Medicine of the U.S. Food and Drug Administration issued a Notice to Industry in July 2009, permitting the use of chromium propionate (CrPro) as a source of chromium supplementation in cattle diets (22). CrPro, an organically bound chromium compound with good chemical stability and solubility, is currently the only source of chromium allowed in cattle diets, with a content as high as 0.5 mg Cr/kg dry matter intake (DMI) (23). Studies have found that CrPro enhances insulin function, has antioxidant and antimicrobial properties, reduces plasma non-esterified fatty acid (NEFA) concentrations, and improves animal growth performance (24). Many studies have shown that CrPro has potential positive effects on health, lactation, rumen microbiota, antioxidation, blood parameters, and innate immunity in periparturient dairy cows (16, 25–28).

Heat stress is a widespread issue in dairy farming, negatively impacting cow welfare, physiological functions, and farm profitability (29). In recent years, the importance of chromium in alleviating heat stress and enhancing production performance in animals has been increasingly recognized. However, studies on the effects of different doses of CrPro on heat-stressed dairy cows are limited. Therefore, this study aimed to conduct a comprehensive analysis through environmental temperature and humidity monitoring, production performance, blood parameters, ruminal fermentation indices, and microbial diversity, which further determines the appropriate dosage of CrPro in practical applications.

## 2 Materials and methods

### 2.1 Experimental design and selection of experimental animals

The experiment was conducted in June 2022 at a large-scale dairy farm in Shaanxi Province, China. Thirty Holstein cows with similar parity (2.3 ± 0.1 parity), days in milk (163.6 ± 2.3), and milk yield (47.0 ± 1.4 kg/d) were randomly assigned to three groups: a control group (Con), a low-dose group (CrL), and a high-dose group (CrH), with 10 cows in each group. The cows were fed diets supplemented with 0, 4, and 8 mg/(d·cow) of CrPro, respectively. The basal diet contained 0.167 mg/kg of chromium. Cows were allowed free access to feed and water, with daily feeding once. The trial lasted for 45 days, preceded by a 15-day adaptation period. The experimental diet was based on a standard lactating cow ration (see Table 1). CrPro was provided as KemTRACETM chromium propionate dry powder by DSM Nutritional Products (Zhuhai) Co., Ltd. The Northwest A&F University Institutional Animal Care and Use Committee approved all procedures involving animals in this study (No. 2021049).

**Table 1:** Test diet composition and nutrient level.

Composition	Proportion	Main ingredients and nutritional indicators	Proportion and content
Corn silage	15.57%	Ca	0.96%
Corn	25.45%	P	0.45%
Alfalfa silage	7.51%	Mg	0.49%
Soybean meal	13.21%	K	1.69%
High quality alfalfa	12.51%	S	0.26%
Whole cotton seed	5.01%	Na	0.54%
Soybean husk	4.00%	Cl	0.55%
Sugar cane molasses	3.03%	CP	18.35%
Double base vegetable	3.01%	ADF	16.68%
Beet granules	2.50%	NDF	26.80%
Expanded soybean	1.50%	EE	4.31%
Corn gluten meal	1.53%	NFC	42.91%
Stone powder	1.11%	Ash	9.56%
Baking soda	1.00%	Fe	18.61 mg/kg
Salt	0.55%	Zn	5.94 mg/kg
Vitamin B	0.55%	Cu	2.26 mg/kg
Potassium carbonate	0.44%	Mn	3.88 mg/kg
Magnesium oxide	0.39%	Se	30 μg/kg
Sodium hydrogen phosphate	0.39%	Co	35 μg/kg
1% lactation premix	0.33%	I	45 μg/kg
Yeast culture	0.26%	Sr	30.87 mg/kg
Methionine	0.11%	ME	2.76 mCal/kg
Demildew agent	0.02%	NEI	1.78 mCal/kg
Probiotics	0.02%	NEG	1.2 mCal/kg
Total	100%	NEM	1.78 mCal/kg

### 2.2 Environmental temperature and humidity index detection

During the experimental period, environmental temperature and humidity sensors were placed at a height of 1.5 m above the ground in the cow barn, following meteorological observation standards. The temperature and relative humidity inside the barn were monitored, with three recordings taken daily at 08:00, 14:00, and 20:00. The average values were calculated and used to determine the temperature-humidity index (THI) using the following formula: THI = 0.81 T + (0.99 T − 14.3) RH + 46.3, where T represents temperature (°C) and RH represents relative humidity (%).

### 2.3 Rectal temperature and respiratory rate detection of dairy cows

Rectal temperature (RT) and respiration rate (RR) of cows in each group were measured once on days 0, 15, 30, and 45 of the trial period. RT was measured using a high-precision veterinary thermometer, while RR was determined by counting the number of thoracoabdominal movements within 1 min.

### 2.4 Milk production and milk composition detection

Milk yield and milk composition were assessed during the trial. At the start of the trial (day 0), the milk yield of cows in each group was recorded using the Afimilk system (AFI-9010B) on the farm. Subsequently, daily milk yield was monitored, and the average milk yield for each group was calculated for the periods of 0–15 days, 16–30 days, and 31–45 days. On days 0, 15, 30, and 45, milk samples (80 mL per cow) were collected from each group and analyzed using a milk component analyzer and a somatic cell counter to determine milk composition (fat, protein, non-fat milk solids, lactose, density, and somatic cell count).

### 2.5 Collection and determination of blood samples

Blood samples were collected from each cow via the caudal vein on days 0, 15, 30, and 45 of the trial period. A total of 10 mL of blood was collected, centrifuged at 3,000 r/min to obtain plasma, and stored at −80°C for subsequent analysis. Biochemical parameters were measured using an automated biochemical analyzer. The parameters included malondialdehyde (MDA, A003-1-2), GSH-Px (A005-1-1), SOD (A001-3-2), alkaline phosphatase (ALP, A059-2-2), aspartate aminotransferase (AST, C010-2-1), alanine aminotransferase (ALT, C009-2-1), glucose (GLU, F006-1-1), total cholesterol (T-CHO, A111-1-1), triglycerides (TAG, A110-1-1), high-density lipoprotein (HDL, A112-1-1), low-density lipoprotein (LDL, A113-1-1), total protein (TP, A045-2-2), albumin (ALB, A028-2-1), urea, calcium (Ca^2+^), sodium (Na^+^), and potassium (K^+^). All measurements were performed according to the instructions provided in the respective assay kits.

### 2.6 Collection of rumen fluid

Rumen fluid was collected on days 0 and 45 of the trial period. A 50 mL sample of rumen fluid was obtained via rumen puncture. Immediately after collection, 5 mL of 25% metaphosphoric acid was added to each sample, which was then vortexed and mixed thoroughly before being aliquoted into 10-mL centrifuge tubes and stored at −20°C for subsequent analysis.

### 2.7 Rumen fermentation parameter analysis

#### 2.7.1 Gas chromatography sample preparation

Three milliliters of sample were placed in a 5-mL centrifuge tube, followed by the addition of 1 mL ultrapure water. The tube was sealed with paraffin film and vortexed for 10 min. Subsequently, 0.15 mL of 50% H₂SO₄ solution was added and mixed thoroughly. Then, 1.6 mL of ether was added, and the tube was inverted 10 times and placed on ice. The mixture was vigorously shaken in a shaker for 20 min, with inversion twice during the process, and this procedure was repeated 10 times. After balancing, the sample was centrifuged at 12,000 rpm for 5 min. One milliliter of the upper ether layer was carefully transferred to a concentration tube, placed on ice, and gently blown with nitrogen gas to reduce the volume to 0.2 mL. The concentrated sample was then transferred to an autosampler vial for injection.

#### 2.7.2 Gas chromatography analysis

Gas chromatography was performed using an Agilent instrument with nitrogen as the carrier gas at a flow rate of 2.0 mL/min and a split ratio of 25:1. The column used was a DB-FFAP capillary column. The temperature program was as follows: initial temperature at 50°C for 3 min, increased to 130°C at a rate of 10°C/min, then to 170°C at 5°C/min, and finally to 220°C at 15°C/min, held for 3 min. The injection temperature was 250°C, and the detector used was a flame ionization detector (FID) with a temperature of 270°C. The injection volume was 2 μL. The parameters measured included acetic acid, propionic acid, butyric acid, valeric acid, isobutyric acid, and isovaleric acid.

### 2.8 Rumen microbiome determination

In this study, the composition and diversity of the rumen microbiota were analyzed using 16S rRNA sequencing (BioProject: PRJNA1296860).

#### 2.8.1 Sequencing sample pretreatment

The appropriate amount of sample (between 0.2–0.5 g) was removed from the refrigerator and added to the centrifuge tube containing the extract lysate for grinding.

#### 2.8.2 Extraction and detection of microbiome total DNA

The microbiome total DNA was extracted by the OMEGA Soil DNA kit (D5625-01, Omega Bio-Tek, Inc.). The extracted DNA was evaluated by 0.8% agarose gel electrophoresis and quantified by Nanodrop (Thermo Fisher Scientific).

#### 2.8.3 The bacterial 16S rRNA gene was amplified by PCR

In this study, sequencing of the V3–V4 hypervariable region (~480 bp) of the bacterial 16S rRNA gene was performed using targeted primers to characterize community-level taxonomic composition and diversity. The primer information of 16S rRNA V3-V4 is shown in Table 2. The barcode in the primer is a 7–10 base oligonucleotide sequence used to distinguish different samples in the same library. ABclonal DNA polymerase (RK20717) was used for PCR. Reaction system: ABclonal DNA polymerase 0.25 μL, 5 × Reaction Buffer 5 μL, 5 × High GC Buffer 5 μL, dNTP (10 mM) 5 μL, template DNA 1 μL, and upstream and downstream primers 1 μL, respectively. Finally, it was supplemented with ddH_2_O to 20 μL. Reaction conditions: predenaturation at 98°C for 5 min, retention at 98°C for 30 s to denature the template, and then the temperature was reduced to 52°C for 30 s to fully anneal the primer and template. Held at 72°C for 45 s, the primer extends on the template, synthesizes DNA, and completes a cycle. This cycle is repeated 25 times to enrich the amplified DNA fragments. Finally, the product was kept at 72°C for 5 min, so that the extension of the product was complete, and the product was preserved at 12°C. The target fragments were extracted by 2% agarose gel electrophoresis, and the target fragments were recovered by the magnetic bead recovery method.

**Table 2:** Primer information of 16S rRNA V3–V4.

Primer name	Sequence information
338F	5′-ACTCCTACGGGAGGCAGCA-3′
806R	5′-GGACTACHVGGGTWTCTAAT-3′

#### 2.8.4 PCR product quantification and sample mixing

Quantification of PCR products on a Microplate reader was performed using the Quant-iT PicoGreen dsDNA assay kit (P7589, Thermo Fisher Scientific), and then mixing of samples were mixed according to the required amount of data for each sample.

#### 2.8.5 Library construction

Illumina’s TruSeq Nano DNA LT Library Prep kit (FC-121-4003, Illumina, Inc.) was used to construct the library. First, End Repair Mix2 in the kit was used to remove the protruding bases at the 5′ end of DNA and complement the missing bases at the 3′ end. At the same time, a phosphate group was added to the 5′ end of DNA. Water 30 ng of the mixed DNA fragments to 60 μL and add 40 μL to End Repair Mix2. Mix well with a gun and incubate at 30°C for 30 min on the PCR apparatus. The end repair system was purified by BECKMAN AMPure XP beads and finally eluted with 17.5 μL Resuspension buffer. The second step is to add A to the 3′ end of the DNA, adding A single A base to the 3′ end of the DNA to prevent the self-joining of the DNA segment, while ensuring that the DNA is connected to the 3′ end of the sequencing splice with a T base protruding. 12.5 μL, A-Tailing Mix was added to the DNA after fragment selection. The mixture was blown with a gun and incubated in a PCR apparatus. The procedure was as follows: 37°C, 30 min; 70°C, 5 min; 4°C, 5 min; held at 4°C. The third step is to add a splice with a specific tag to allow the DNA to eventually hybridize to the Flow Cell. In the system with A added, 2.5 μL Resuspension buffer, 2.5 μL Ligation Mix, and 2.5 μL DNA adapter Index were added. The mixture was mixed with a gun and placed on a PCR apparatus and incubated at 30°C for 10 min. The 5 μL Stop Ligation buffer was added. The system was purified by BECKMAN AMPure XP beads. The fourth step is to amplify the spliced DNA by PCR and then purify the PCR product using BECKMAN AMPure XP beads. The fifth step is the final fragment selection and purification of the library by 2% agarose gel electrophoresis.

#### 2.8.6 Quality inspection and sequencing

A 1-μL aliquot of the library was subjected to quality control using the Agilent high-sensitivity DNA kit (5067-4626, Agilent high sensitivity DNA kit) on the Agilent Bioanalyzer 2100 (G2939A, Agilent Technologies, Inc.). A qualified library should exhibit a single peak without adapter dimers. The concentration of the library was determined using the Quant-iT PicoGreen dsDNA assay kit (P7589, Thermo Fisher Scientific) on the Promega QuantiFluor instrument. A qualified library should have a concentration of at least 2 nM. Qualified libraries were sequenced on the Illumina NovaSeq platform using the NovaSeq 6000 SP reagent kit (20028402, Illumina, Inc.) (500 cycles) for 2 × 250 bp paired-end sequencing. Libraries with unique indices were first diluted to 2 nM and then pooled in proportion to the desired sequencing depth. The pooled libraries were denatured with 0.1 N NaOH to generate single-stranded DNA for sequencing. The loading concentration of the libraries was adjusted to 15–18 pM based on experimental requirements.

#### 2.8.7 Statistical analysis of microbiome data

The microbiome information analysis process involves the use of QIIME2 software for sequence data processing, including decoding, primer removal, and mass filtering, followed by the use of DADA2 for quality control, denoising, and splicing. The processed sequences were merged into ASVs according to 100% similarity, and the classification status was identified. Next, rare ASVs with an abundance of less than 0.001% were removed, and the removed abundance matrix was used for subsequent analysis. R software was used to draw a bar chart to show the classification and identification results of the samples. QIIME2 was used for Alpha diversity index analysis, a sparse curve was drawn to evaluate sequencing depth, a diversity index was calculated, and a box plot was drawn to compare diversity among samples. Taxonomic composition analysis was performed to check the microbial composition of each sample at various taxonomic levels and to compare the number of microbial groups between samples. Finally, R software is used to draw a bar chart to compare the number of taxon in different samples at the same level.

### 2.9 Data processing and statistical analysis

All data were analyzed using GraphPad Prism 8.0.2 software. One-way ANOVA was employed for statistical analysis. Results are expressed as the mean ± standard error of the mean (SEM). Differences were considered non-significant when *p* > 0.05 and significant when *p* < 0.05.

## 3 Results

### 3.1 Monitoring of temperature-humidity index in cattle barns

**Figure 1:**
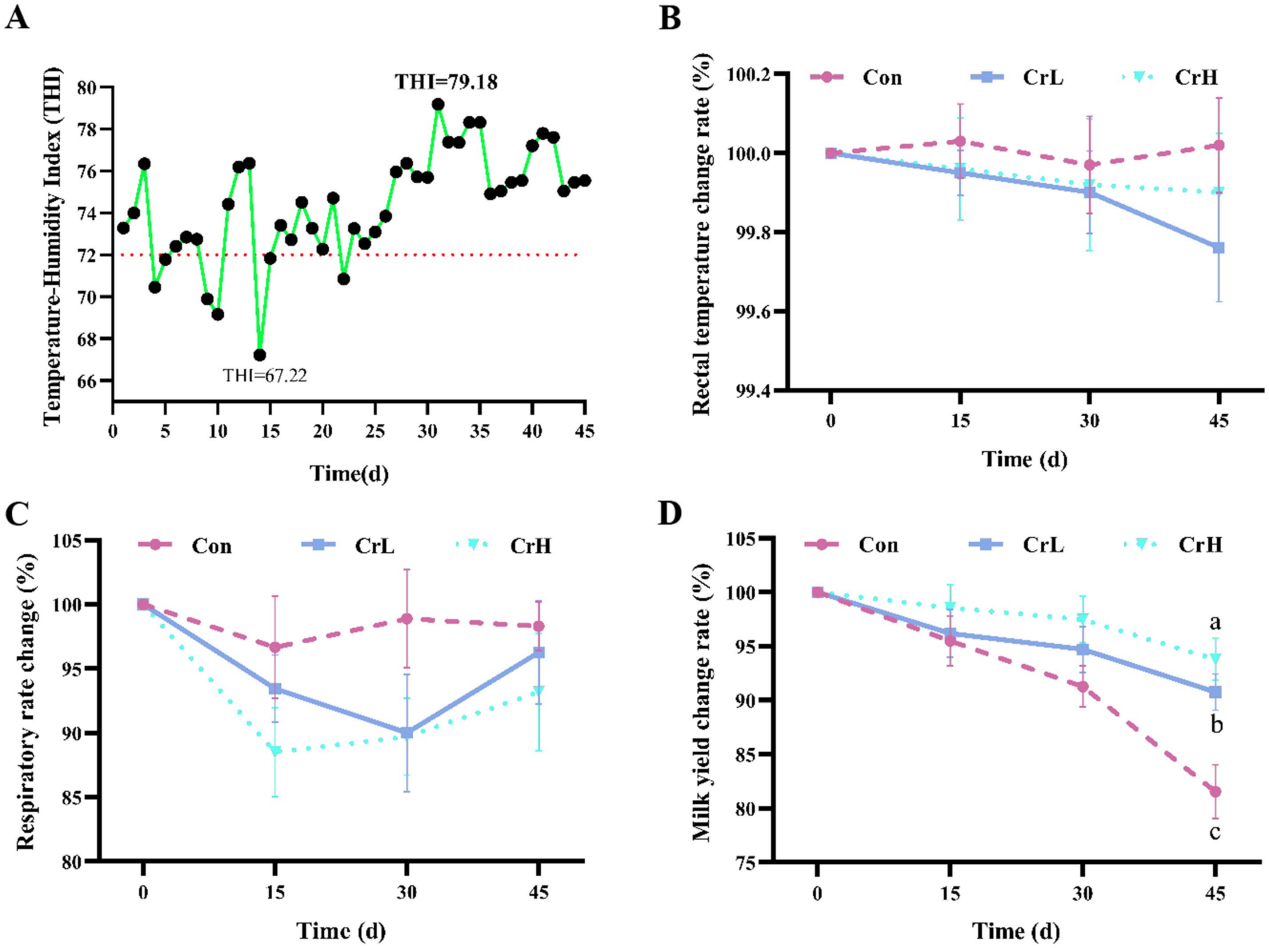
Heat stress index and milk yield detection. THI variation curve of each group in the experiment period. THI values exceeding 72 indicate that the cows were experiencing mild heat stress **(A)**. Effect of different concentrations of CrPro on rectal temperature **(B)**, respiratory rate **(C)** and milk yield **(D)** in dairy cows. Results are expressed as the mean ± standard error of the mean (SEM). Different lowercase letters indicate significant differences between treatments (*p* < 0.05).

THI is an important indicator for assessing the impact of heat stress on dairy in barn environments (30). As shown in Figure 1A, with the advancement of the trial days, the THI shows an increasing trend. During the experimental period (0–45 days), the minimum THI in the barns was 67.22 (on day 14), and the maximum THI was 79.18 (on day 31), with an average value of 74.97 ± 2.85. A THI value greater than 72 indicates that dairy cows were experiencing mild heat stress.

### 3.2 Effects of dietary CrPro on rectal temperature and respiratory rate of dairy cows

As shown in Figures 1B,C, compared with the Control group, RT of dairy cows in the CrL and CrH groups tended to decrease. Compared with the Con group, RR of dairy cows in the CrL and CrH groups decreased at all time points (15, 30, and 45 days), although the differences were not significant (*p* > 0.05).

### 3.3 Effects of dietary CrPro on milk yield in dairy cows

As shown in Figure 1D, milk yield in all groups decreased with the prolonged duration of heat stress. Compared with the Control group, the decline was more gradual in the CrL and CrH groups. During days 31–45, milk yield was significantly higher in the CrL group than in the Con group (*p* = 0.0278) and significantly higher in the CrH group than in the Con group (*p* = 0.0031). As shown in Table 3, in terms of total milk yield, compared with milk yield at the start of the trial (day 0), at day 45, the Con group lost 8.27 kg of milk per cow per day, corresponding to an economic loss of CNY 33.08; the CrL group lost 4.11 kg of milk per cow per day, corresponding to an economic loss of CNY 16.44; and the CrH group lost 2.76 kg of milk per cow per day, corresponding to an economic loss of CNY 11.04. Compared with the Con group, the CrH group reduced economic losses by CNY 22.04.

**Table 3:** Effect of different concentrations of CrPro on milk yield of dairy cows.

Time (d)	Average milk yield (kg)		
	Con	CrL	CrH
0	44.78	44.52	44.35
5	42.76	42.82	43.71
15	40.87	42.17	43.22
45	36.51	40.41	41.59
Decreasing quantity	8.27	4.11	2.76

### 3.4 Effects of dietary CrPro on milk composition in dairy cows

**Figure 2:**
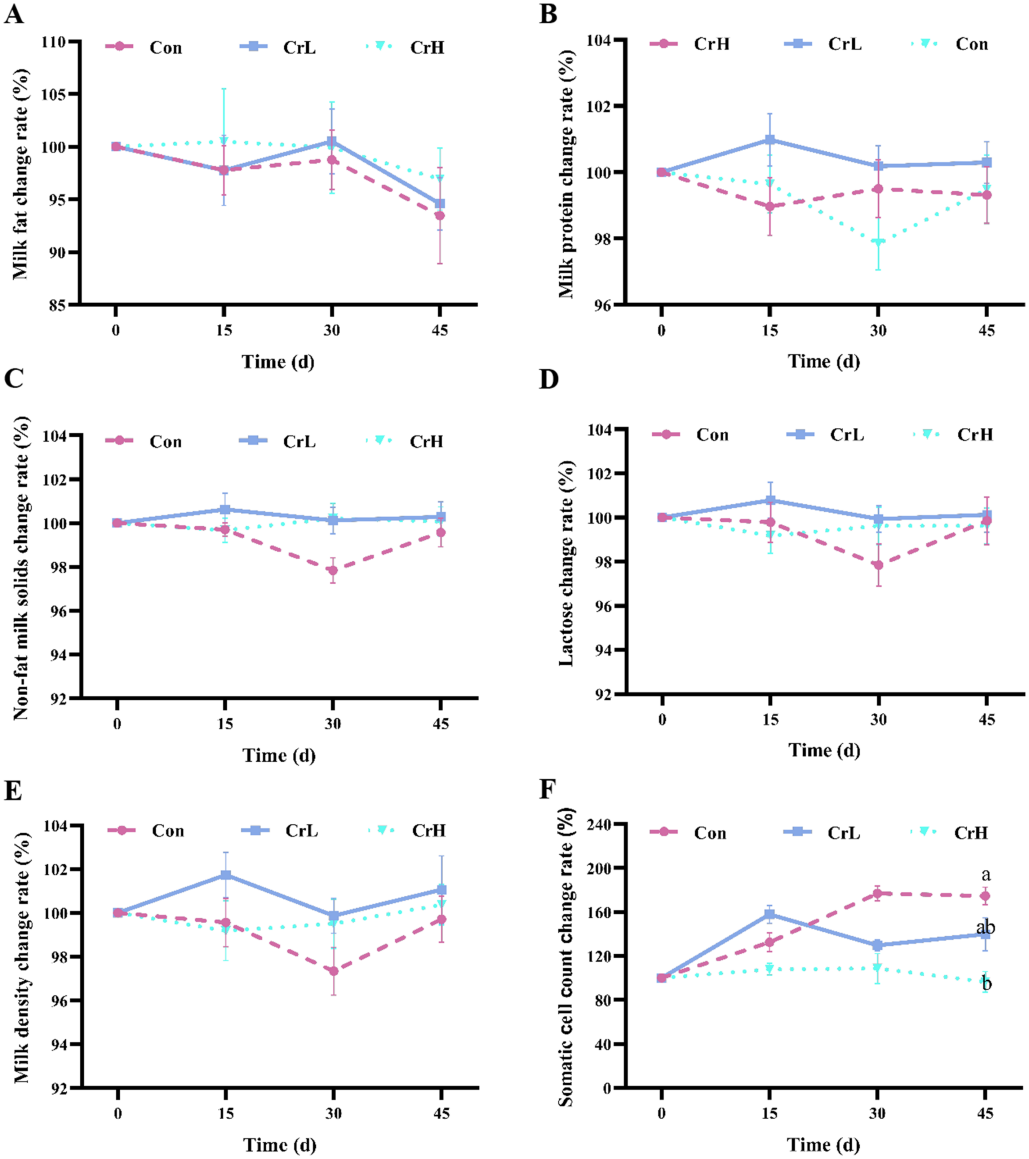
Effects of dietary CrPro on milk composition in dairy cows. Detection of milk fat **(A)**, milk protein **(B)**, non-fat milk solids **(C)**, lactose **(D)**, milk density **(E)**, somatic cell count **(F)** change rate. Results are expressed as the mean ± standard error of the mean (SEM). Different lowercase letters indicate significant differences between treatments (*p* < 0.05).

As shown in Figure 2A, compared with the start of the trial (0 days), milk fat content decreased in all three groups at the end of the trial (45 days). No significant differences in milk fat content were observed between the CrL and CrH groups and the Con group (*p* > 0.05). As shown in Figures 2B–E, the changes in milk protein, non-fat milk solids, lactose, and density were similar between the CrL and CrH groups, with no significant differences compared with the Con group (*p* > 0.05). As shown in Figure 2F, the somatic cell count (SCC) in milk of the Con group increased over the trial period, whereas SCC in the CrH group remained stable. At 45 days, SCC in the CrH group was significantly lower than that in the Con group (*p* < 0.05).

### 3.5 Effects of dietary CrPro on antioxidant indicators and hepatic enzyme activity in dairy cows

**Figure 3:**
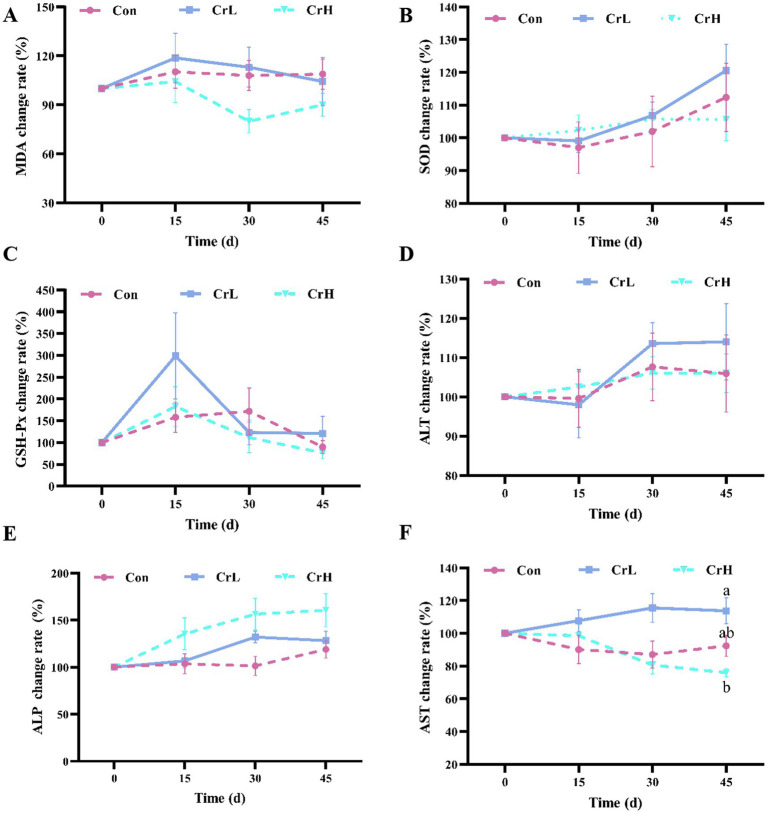
Effects of dietary CrPro on antioxidant indicators and hepatic enzyme activity in dairy cows. Detection of MDA **(A)**, SOD **(B)**, GSH-Px **(C)**, ALT **(D)**, ALP **(E)**, AST **(F)** content change rate. Results are expressed as the mean ± standard error of the mean (SEM). Different lowercase letters indicate significant differences between treatments (*p* < 0.05).

As shown in Figure 3, no significant differences in MDA were observed among groups (*p* > 0.05). However, compared with the Con group, MDA levels in the CrH group were reduced at all time points (15, 30, and 45 days) during the trial (Figure 3A). No significant differences in SOD activity were detected among groups (*p* > 0.05), but SOD activity in the Con and CrL groups tended to increase, whereas the CrH group remained relatively stable (Figure 3B). Changes in GSH-Px activity were similar among groups, with no significant differences observed (*p* > 0.05) (Figure 3C). No significant differences in ALT activity were detected among groups (*p* > 0.05) (Figure 3D). The ALP in the CrH group was slightly higher than that in the CrL group and the Con group, but there was no significant difference (*p* > 0.05) (Figure 3E). At day 45, AST activity in the CrL group was significantly higher than that in the CrH group (*p* < 0.05), but no significant differences were observed between the CrH and CrL groups and the Con group (*p* > 0.05) (Figure 3F).

### 3.6 Effects of dietary CrPro on lipid metabolism, glucose metabolism and nitrogen metabolism in dairy cows

**Figure 4:**
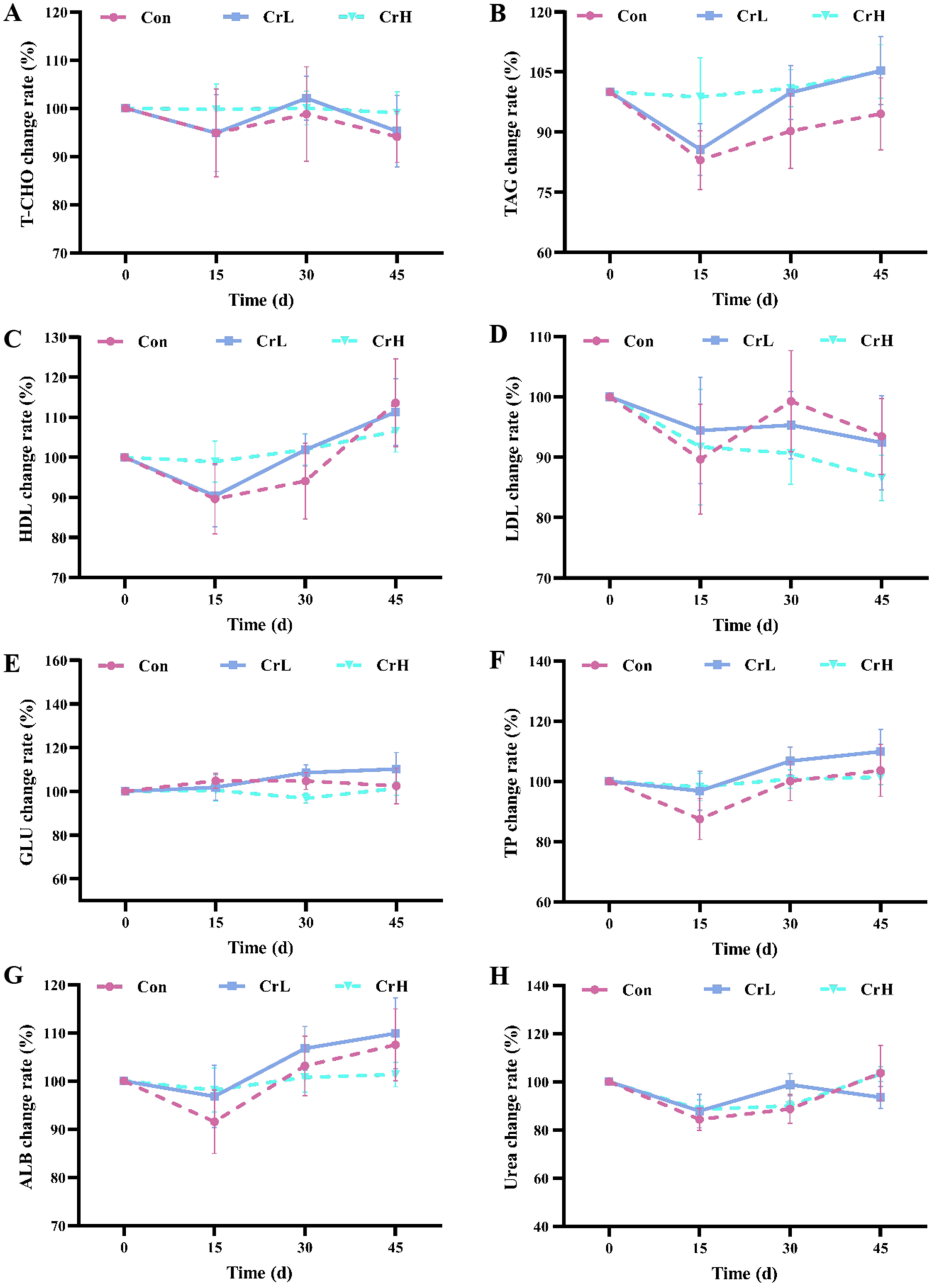
Effects of dietary CrPro on lipid metabolism, glucose metabolism and nitrogen metabolism in dairy cows. Detection of T-CHO **(A)**, TAG **(B)**, HDL **(C)**, LDL **(D)**, GLU **(E)**, TP **(F)**, ALB **(G)**, urea **(H)** content change rate.

As shown in Figure 4, T-CHO in the Con and CrL groups fluctuated within the normal range, while T-CHO in the CrH group remained stable (Figure 4A). TAG and HDL in the Con and CrL groups showed a downward trend from 0 to 15 days and an upward trend from 15 to 45 days, whereas these parameters remained stable in the CrH group (Figures 4B,C). LDL in the Con and CrL groups exhibited a downward trend from 0 to 15 days, an upward trend from 15 to 30 days, and a downward trend from 30 to 45 days, while LDL in the CrH group showed a continuous downward trend over the entire 45-day period (Figure 4D). No significant differences were observed among groups for T-CHO, TAG, HDL, and LDL (*p* > 0.05). Additionally, GLU remained stable in all groups (Figure 4E). TP and ALB showed similar trends, with a downward trend from 0 to 15 days and upward trends from 15 to 30 days and from 30 to 45 days (Figures 4F,G). Urea exhibited minor fluctuations in all groups (Figure 4H). No significant differences were observed among groups for GLU, TP, ALB, and urea (*p* > 0.05).

### 3.7 Effects of dietary CrPro on blood electrolytes in dairy cows

**Figure 5:**
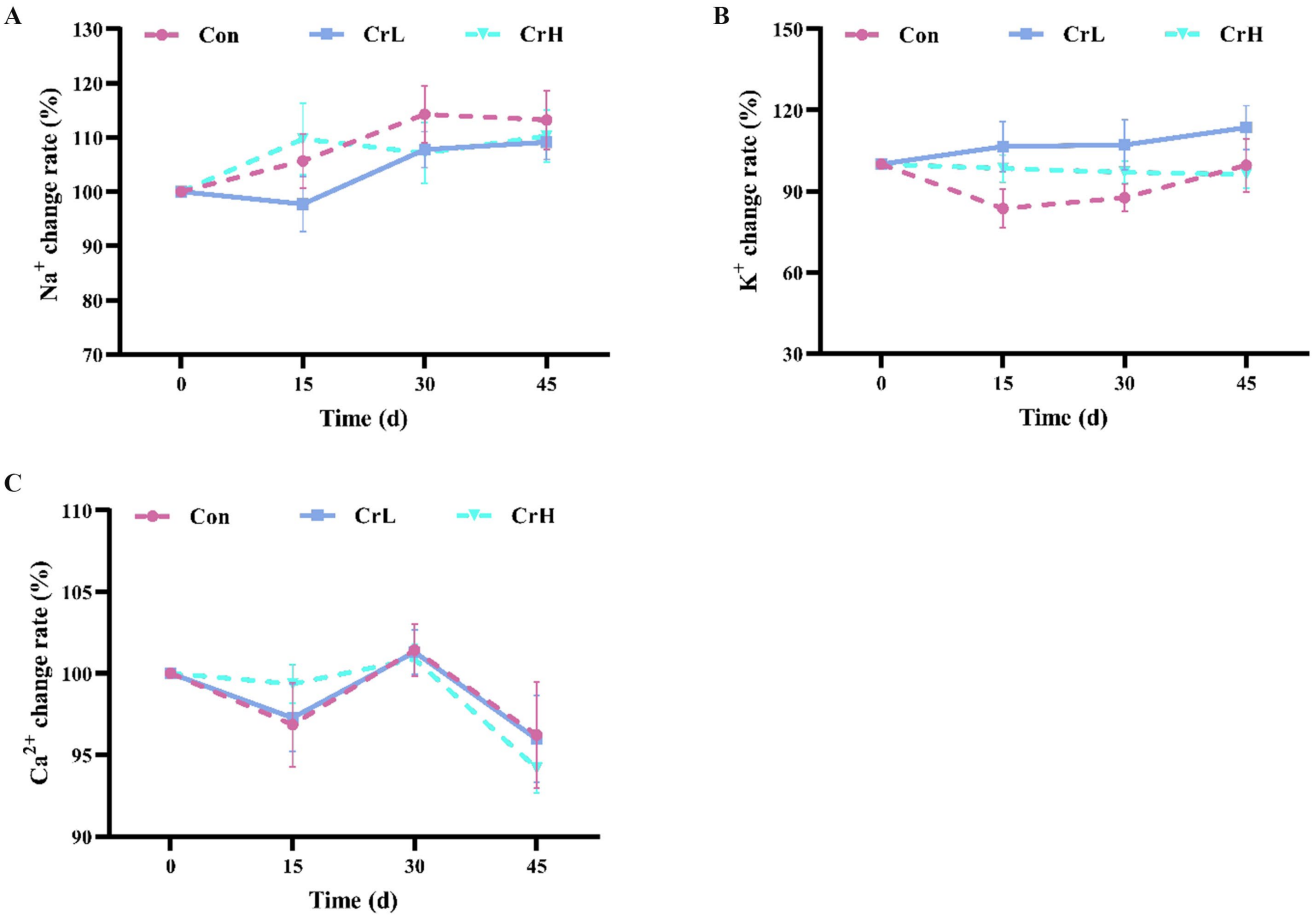
Effects of dietary CrPro on blood electrolytes in dairy cows. Detection of Na^+^ **(A)**, K^+^ **(B)**, Ca^2+^ **(C)** content change rate.

As shown in Figure 5, calcium (Ca^2+^) levels in all groups exhibited a downward trend from 0 to 15 days, an upward trend from 15 to 30 days, and another downward trend from 30 to 45 days. Potassium (K^+^) levels in the CrL group showed an upward trend from 0 to 45 days, while K^+^ levels in the CrH group exhibited a downward trend over the same period. Sodium (Na^+^) levels in all groups fluctuated within a certain range but generally showed an upward trend. No significant differences were observed among groups for Na^+^, K^+^, and Ca^2+^ (*p* > 0.05).

### 3.8 Effects of dietary CrPro on rumen fermentation parameters in heat-stressed dairy cows

**Figure 6:**
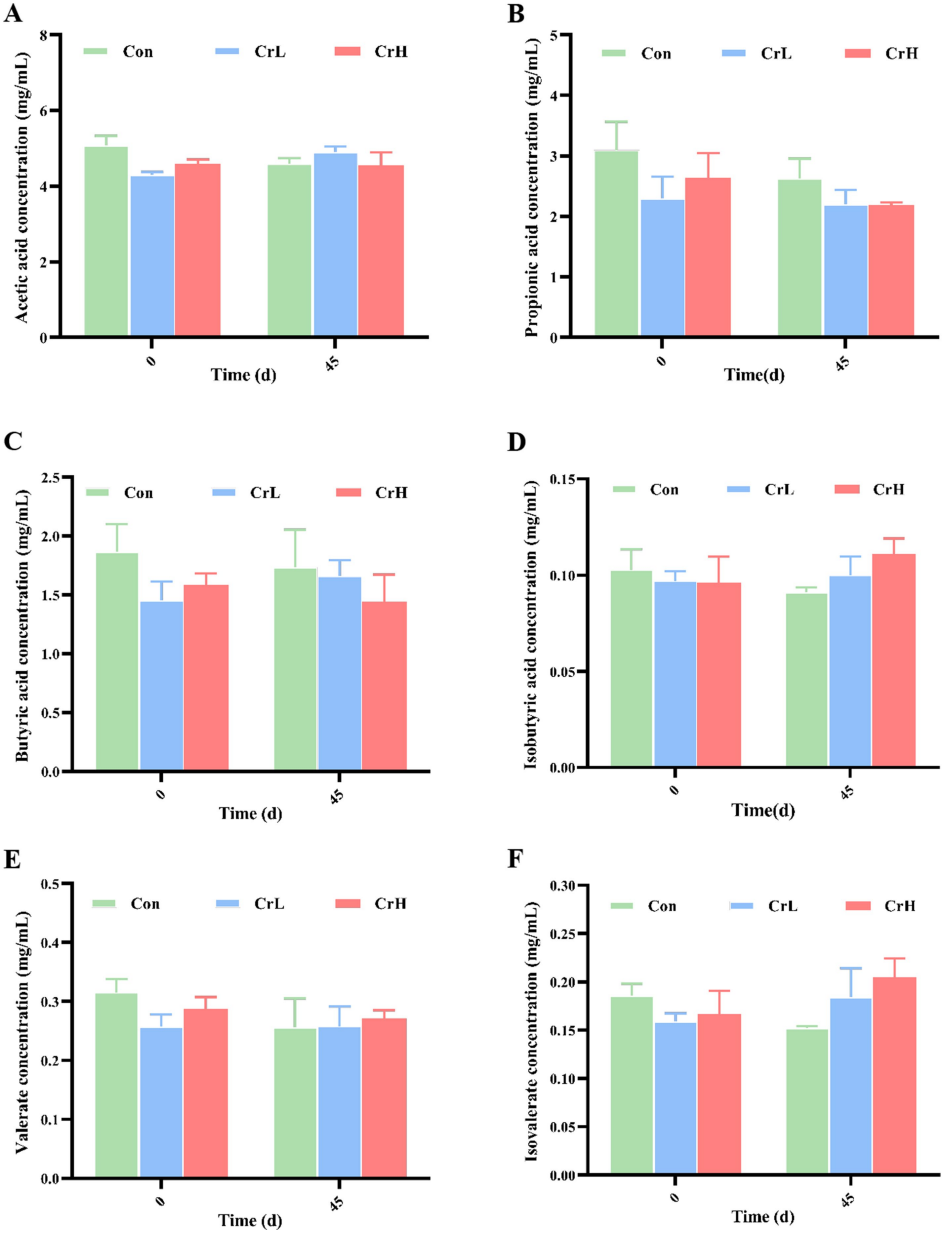
Effects of dietary CrPro on rumen fermentation parameters in heat-stressed dairy cows. Detection of acetic acid **(A)**, propionic acid **(B)**, butyric acid **(C)**, isobutyric acid **(D)**, valerate acid **(E)**, isovalerate acid **(F)** content change rate.

As shown in Figure 6, compared with day 0 of the trial, total VFAs in the Con group decreased at day 45 (*p* > 0.05). In the CrL group, propionate concentration decreased, while concentrations of other VFAs increased (*p* > 0.05). In the CrH group, acetate concentration remained stable, while concentrations of propionate, butyrate, and valerate decreased (*p* > 0.05), whereas isobutyrate and isovalerate concentrations increased (*p* > 0.05).

### 3.9 Effects of dietary CrPro on rumen microbial diversity in heat-stressed dairy cows

**Figure 7:**
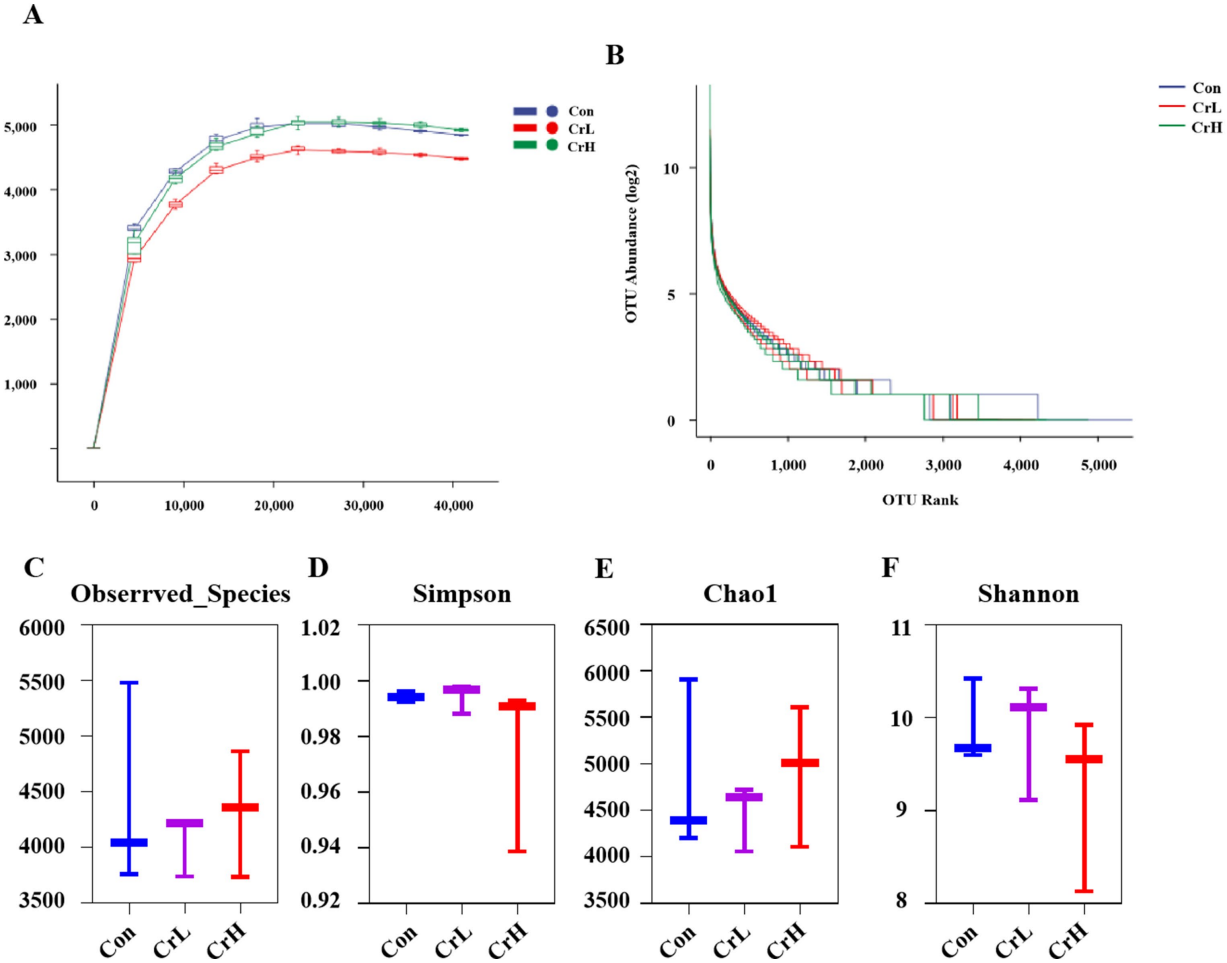
Effects of dietary CrPro on rumen microbial diversity in heat-stressed dairy cows. **(A)** Ruminal bacterial rarefaction curves. **(B)** Rumen bacteria abundance rating curve. **(C–F)** Effect of different concentrations of CrPro on various microbial diversity indices in dairy cows.

Rumen microbiota plays a crucial role in the health, growth, and reproduction of dairy cows (31). The results of the study on rumen microbiota in heat-stressed dairy cows supplemented with CrPro are shown in Figure 7. The rarefaction curves (Figure 7A) and rank abundance curves (Figure 7B) of rumen microbial species show a stable trend, indicating that the sequencing depth and quantity were sufficient to cover the species present from the samples and meet the requirements for subsequent analyses. Additionally, compared with the Con group, the Chao1, and Observed species indices showed an increasing trend with increasing CrPro supplementation levels, although differences were not significant (*p* > 0.05). No significant differences were observed among groups for the Shannon and Simpson indices (*p* > 0.05).

**Figure 8:**
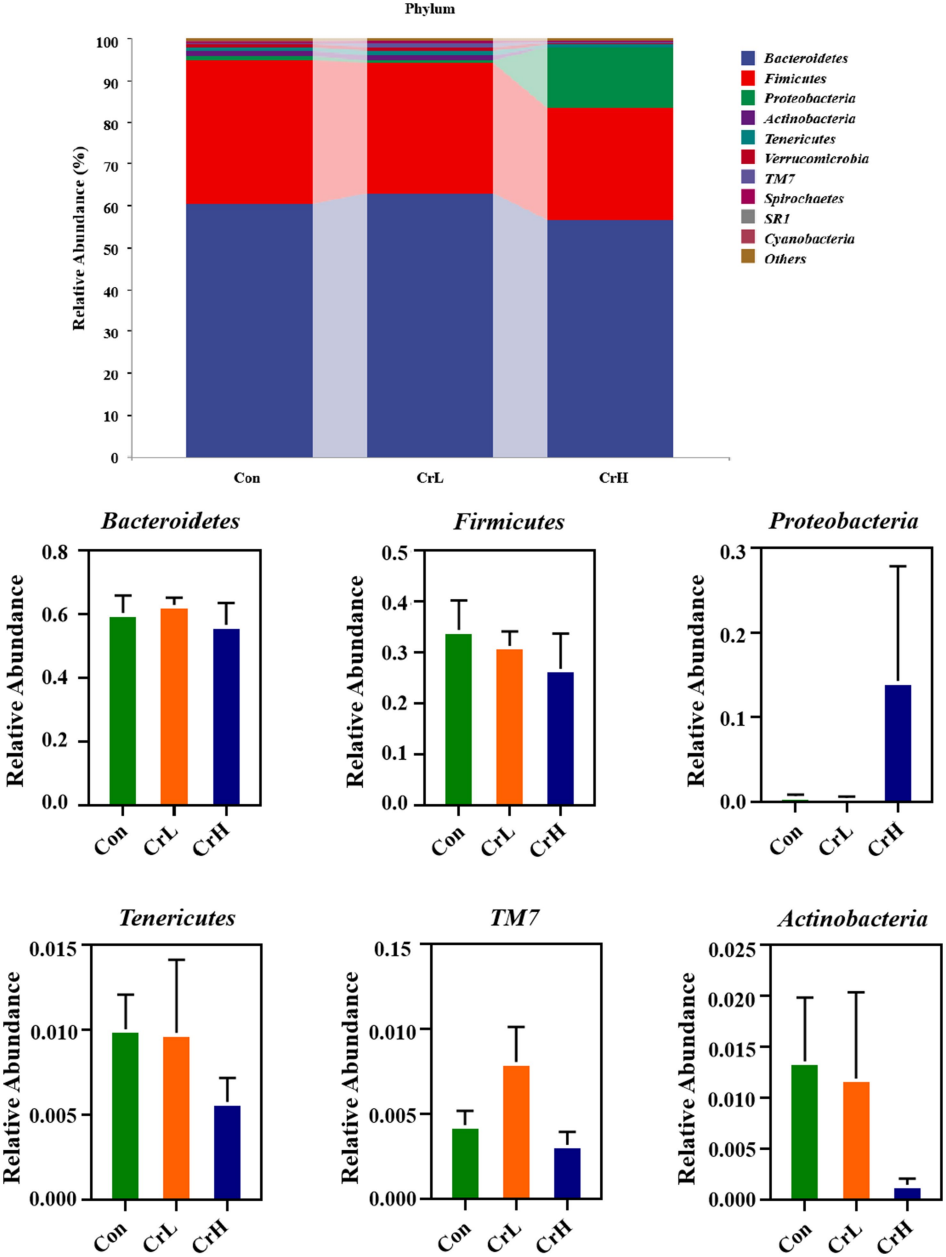
Effects of different concentrations of CrPro on rumen microbial composition at the phylum in dairy cows.

The effects of dietary CrPro on the rumen microbial composition at the phylum and genus levels in heat-stressed dairy cows were investigated. Through species composition analysis, the top 10 most abundant phyla and the top 20 most abundant genera were selected for rumen microbial profiling, as shown in Figure 8. At the phylum level, the control group (Con) had the following dominant phyla: Bacteroidota (60.34%), *Firmicutes* (34.39%), *Actinobacteria* (1.36%), *Tenericutes* (1.01%), and *Proteobacteria* (0.72%). The chromium low-dose group (CrL) had *Bacteroidota* (62.88%), *Firmicutes* (31.26%), *Actinobacteria* (1.19%), *Tenericutes* (0.98%), and *Proteobacteria* (0.49%). The chromium high-dose group (CrH) had *Bacteroidota* (56.64%), *Firmicutes* (26.76%), *Proteobacteria* (14.29%), and *Tenericutes* (0.58%). The *Firmicutes*-to-*Bacteroidota* (F/B) ratios were 0.56, 0.49, and 0.47 for the Con, CrL, and CrH groups, respectively. No significant differences were observed among groups at the phylum level (*p* > 0.05).

**Figure 9:**
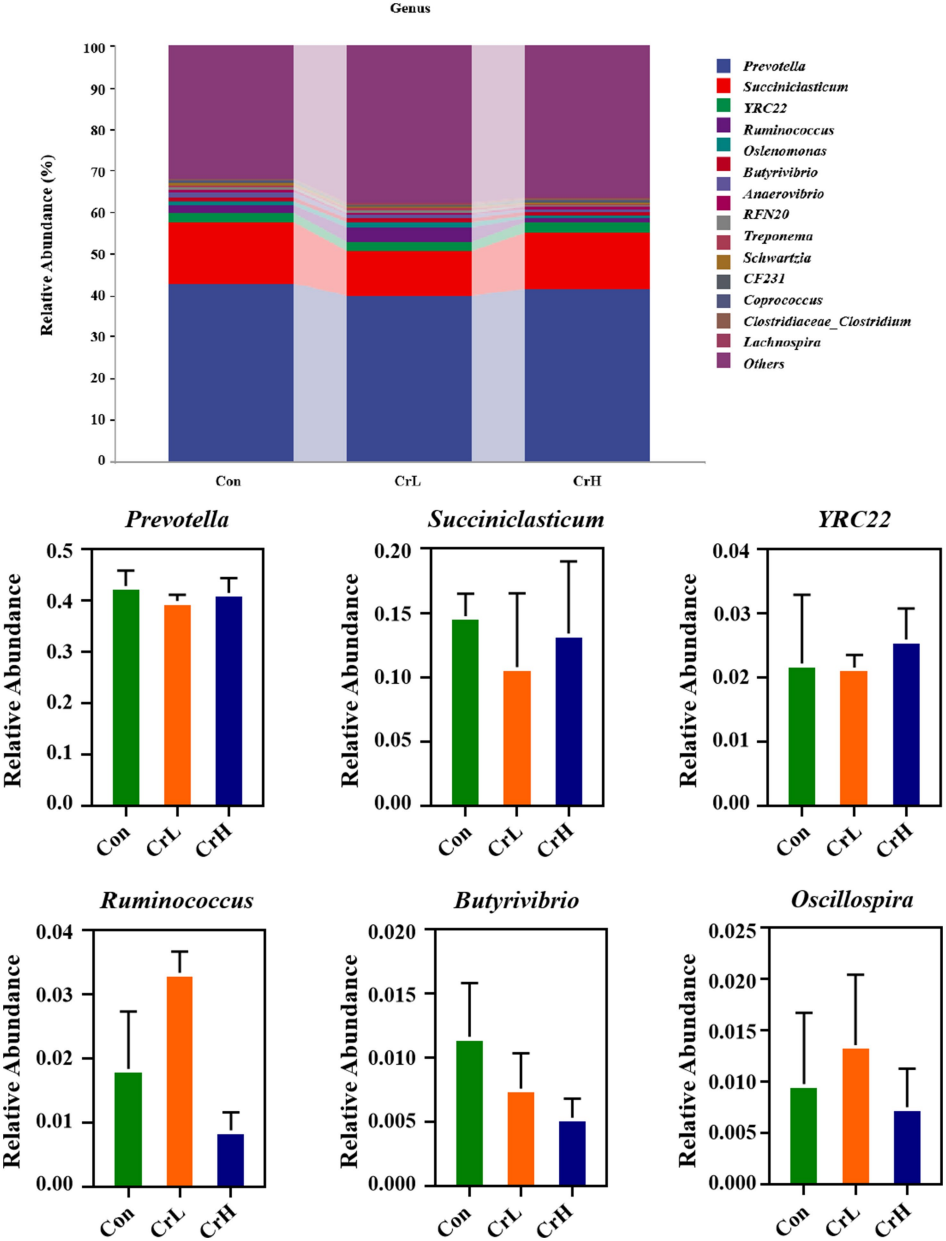
Effects of different concentrations of CrPro on rumen microbial composition at the genus in dairy cows.

As shown in Figure 9, at the genus level, the Con group had the following dominant genera: *Prevotella* (42.79%), *Succiniclasticum* (14.76%), *YRC22* (2.22%), *Ruminococcus* (1.83%), and *Butyrivibrio* (1.16%). The CrL group had Prevotella (39.91%), *Succiniclasticum* (10.78%), *YRC22* (2.15%), *Ruminococcus* (3.33%), *Oscillospira* (1.35%), and *Selenomonas* (1.01%). The CrH group had Prevotella (41.48%), *Succiniclasticum* (13.42%), *YRC22* (2.59%), and *Selenomonas* (1.03%). No significant differences were observed among groups at the genus level (*p* > 0.05).

### 3.10 Correlation analysis of rumen fermentation parameters with lactation performance, blood biochemical indicators, and rumen microbial communities

**Figure 10:**
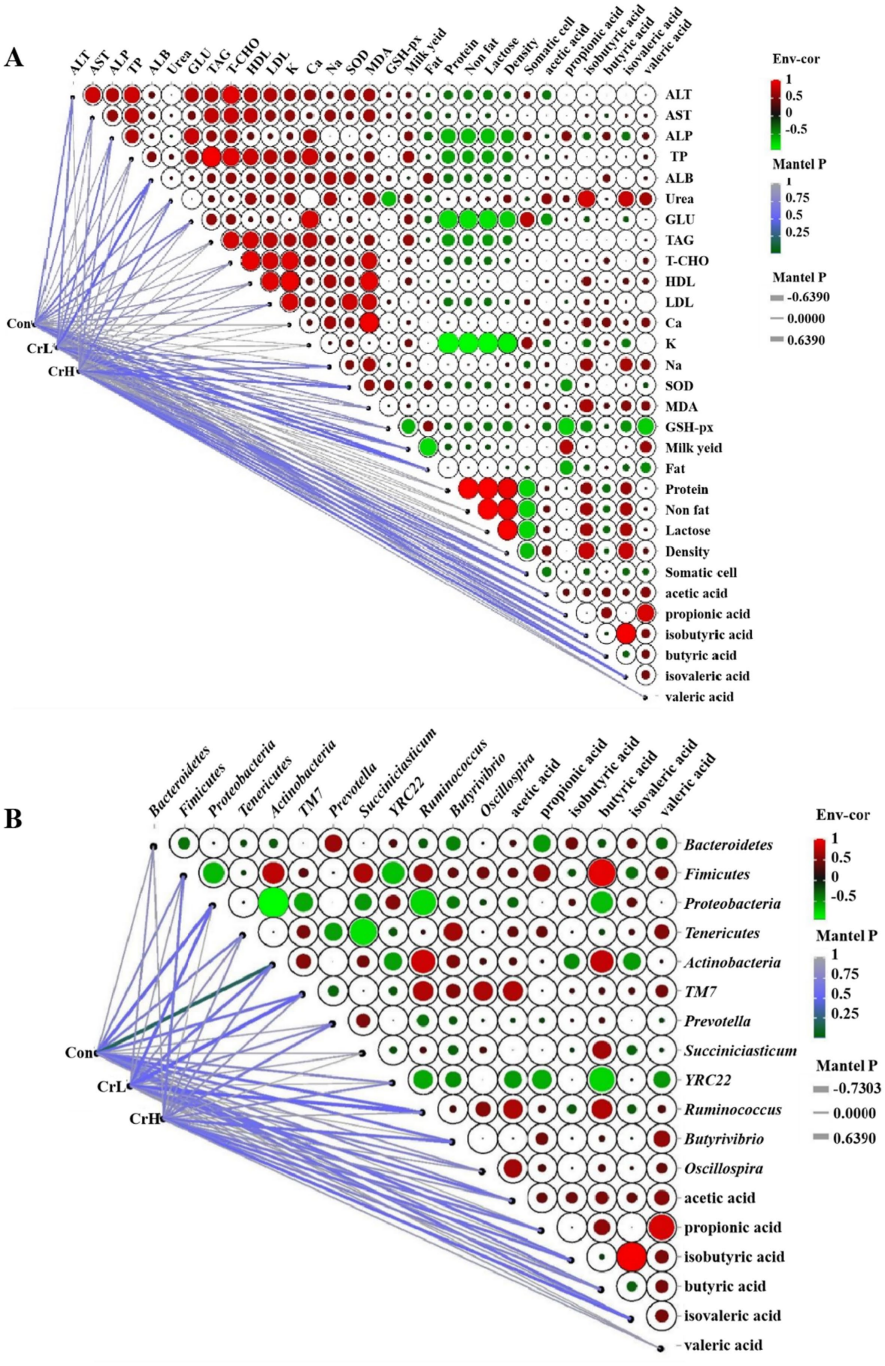
Correlation analysis of rumen fermentation parameters with lactation performance, blood biochemical indicators, and rumen microbial communities. **(A)** Correlation analysis of rumen fermentation parameters with lactation performance and blood biochemical indexes. **(B)** Correlation analysis of rumen microbial flora and rumen fermentation parameters.

As shown in Figure 10A, Pearson correlation analysis was used to assess the relationships between rumen fermentation parameters and lactation performance as well as blood biochemical indicators. The results revealed that propionate was significantly positively correlated with milk yield (*p* < 0.05) and significantly negatively correlated with milk fat and GPX (*p* < 0.05). Isobutyrate was significantly positively correlated with urea content (*p* < 0.05) and showed positive correlations with MDA, milk protein, non-fat, and lactose contents, although these correlations were not significant (*p* > 0.05). Isovalerate was significantly positively correlated with urea content (*p* < 0.05) and showed positive correlations with MDA, milk protein, non-fat, and lactose contents, which were not significant (*p* > 0.05). Butyrate was positively correlated with urea, MDA, and milk yield, and negatively correlated with milk fat, but none of these correlations reached significance (*p* > 0.05).

As shown in Figure 10B, Pearson correlation analysis was used to evaluate the relationships between rumen microbial communities and rumen fermentation parameters. At the phylum level, *TM7* was significantly positively correlated with acetate content (*p* < 0.05). *Bacteroidetes* were significantly negatively correlated with propionate content (*p* < 0.05), while *Firmicutes* were significantly positively correlated with propionate content. *Firmicutes* and *Actinobacteria* were significantly positively correlated with butyrate content (*p* < 0.05), whereas *Proteobacteria* was significantly negatively correlated with butyrate content (*p* < 0.05). *Actinobacteria* was significantly negatively correlated with isovalerate content (*p* < 0.05). At the genus level, *Succiniclasticum* was significantly positively correlated with butyrate content (*p* < 0.05). *YRC22* was significantly negatively correlated with acetate, propionate, butyrate, and valerate contents (*p* < 0.05). *Ruminococcus* was significantly positively correlated with acetate and butyrate contents (*p* < 0.05), and *Oscillospira* was significantly positively correlated with acetate content (*p* < 0.05).

## 4 Discussion

This study investigated the effects of dietary CrPro supplementation on milk yield and milk composition, blood antioxidant and liver enzyme activities, carbohydrate and lipid metabolism, and rumen microbiota diversity in dairy cows under mild heat stress. Compared with the control group, the addition of 4 and 8 mg/(d·cow) CrPro to the basal diet reduced RT and RR in heat-stressed cows and increased milk yield. The 8 mg/(d·cow) CrPro dose also decreased the accumulation of MDA in cows under heat stress, thereby alleviating oxidative stress damage. Additionally, the inclusion of 4 and 8 mg/(d·cow) CrPro enhanced the abundance of rumen microbiota, mitigating the negative impacts of heat stress on rumen microbial communities.

### 4.1 The effects of dietary CrPro on the main physiological signs and production performance of heat-stressed dairy cows

Elevated core body temperature is a direct physiological response to heat stress, which can be monitored via RT measurements. An increase in core body temperature indicates a disruption in the heat production-dissipation balance in dairy cows, negatively affecting various physiological functions (32). RR is considered one of the most sensitive and useful physiological indicators of heat stress. During heat stress, an increase in RT is accompanied by an elevated RR, which facilitates heat dissipation from the body to the environment. During heat stress, changes in RR precede changes in other physiological variables (RT, sweating, pulse, or heart rate) (33). Under hot conditions, RR increases by 2.8–3.3 breaths/min for every 1°C rise in ambient temperature (34). Spires et al. (35) demonstrated that when cows were rapidly transitioned from 19°C to 29°C, their RT and RR significantly increased on the day of transition. In this study, heat stress did not significantly increase RT and RR in cows, possibly because the heat stress state was naturally developed rather than artificially induced by rapid heat load. Compared with the control group, the addition of 4 and 8 mg/(d·cow) of CrPro to the basal diet reduced RT and RR in heat-stressed cows, potentially due to the alleviation of oxidative stress damage by CrPro. Further studies are needed to explore the specific mechanisms.

Studies have shown that milk yield losses due to heat stress in China range from 0.7 to 4.0 kg/(d·cow), with projected losses increasing to 1.5–6.5 kg/(d·cow) by 2050 and 2.0–7.2 kg/(d·cow) by 2070 (36). Milk yield is an important indicator of dairy cow production performance. Under heat stress conditions, heat-sensitive mammary cells are damaged, resulting in reduced numbers and decreased activity of mammary epithelial cells, which directly impacts milk yield (37). Additionally, heat stress alters whole-body metabolism, increasing tissue glucose uptake and limiting glucose supply and lactose synthesis in the mammary gland (38). In addition, the decrease of DMI caused by heat stress is one of the important factors leading to the decrease of milk production. Compared with normal cows, heat stressed cows have decreased DMI, decreased mammary gland blood flow, and greatly reduced nutrients available for mammary gland utilization (39). It has been shown that when the average THI exceeds 68, heat stress will negatively affect the milk production of lactating cows, and the milk production will decrease by 0.2 kg for every 1 increase in THI (40).

Milk composition is an important indicator for assessing milk quality; however, whether chromium affects milk protein, milk fat, and lactose content remains inconclusive. A study by Hayirli et al. (41) showed that the addition of Cr-Met (chromium-methionine) has a quadratic relationship with the increase in milk fat and lactose content. Besong et al. (42) demonstrated that the effect of dietary chromium supplementation is related to the parity of dairy cows. Adding an appropriate amount of chromium to the diet of first-lactation cows can increase milk yield and dry matter intake, as well as enhance milk fat, lactose, and total solids content, while no significant effect was observed in multiparous cows. Study found that Cr supplementation interacted with time, increasing the yields of milk, milk protein, and milk lactose during the CO period (26). Results from this study indicate that supplementing the basal diet of lactating cows with 8 mg/(d·cow) of CrPro can mitigate the decline in milk yield in heat-stressed cows to some extent, 4 and 8 mg/(d·cow) of CrPro had no significant effect on milk composition, which is inconsistent with the findings of Hayirli et al. (41). This discrepancy may be attributed to differences in the intensity of heat stress and the physiological status of the cows. Summer is a high-incidence season for mastitis in dairy cows, and somatic cell count (SCC) is an important indicator for assessing mastitis in cows (43). Results from this study show that adding 8 mg/(d·cow) of CrPro significantly reduced SCC, playing a positive role in the prevention and control of mastitis.

### 4.2 The effects of dietary CrPro on the main physiological signs and production performance of heat-stressed dairy cows

Studies have shown that CrPro may influence blood parameters in dairy cows, particularly under heat stress conditions. Heat stress is a metabolic disorder that disrupts the normal metabolism of dairy cows by increasing intracellular ROS levels, thereby affecting their physiological, hormonal, and immune states (32). Research indicates that during heat stress, plasma MDA levels rise, while the activities of SOD and GSH-Px decrease (44). In this study, cows in the CrH exhibited a trend of reduced MDA accumulation in the body. In contrast, the Con group and the CrL group showed an increasing trend in SOD activity under heat stress. This suggests that supplementing 8 mg/(d·cow) of CrPro can enhance the antioxidant capacity of dairy cows under heat stress and mitigate oxidative stress. The inconsistency with previous studies may be due to compensatory responses in the body under high-temperature stress, such as increasing antioxidant enzyme activity and catalase activity to reduce heat stress-induced cellular damage and maintain normal function.

Studies have shown that during heat stress, dairy cows secrete large amounts of glucocorticoids to maintain the energy requirements for lactation. This leads to increased glucose catabolism, mobilization of body fat, and enhanced protein metabolism (44). As a result, concentrations of GLU, TAG, ALB, and urea increase, while TP concentration decreases (45). Results from this study indicated that supplementing the basal diet of lactating cows with 4 and 8 mg/(d·cow) of CrPro had no significant effect on carbohydrate, lipid, and protein metabolism in heat-stressed cows. This lack of effect may be related to differences in lactation stage and chromium dosage. Additionally, compared with the Con group, there were no significant differences in the activities of ALP, ALT, and AST in the CrL and CrH groups, indicating that supplementing the basal diet of lactating cows with 4 and 8 mg/(d·cow) of CrPro had no significant effect on hepatic enzyme activities in cows.

Heat stress can affect water and mineral metabolism in dairy cows to some extent. During heat stress, the intake of K^+^, Ca^2+^, and Na^+^ is relatively insufficient (46). Results from this study indicate that supplementing the basal diet of lactating cows with 4 and 8 mg/(d·cow) of CrPro had no significant effect on K^+^, Ca^2+^, and Na^+^ levels in cows. This lack of effect may be related to the degree of heat stress, the dietary structure, and the fact that the alleviation of heat stress by CrPro may not involve the regulation of K^+^, Ca^2+^, and Na^+^.

### 4.3 The effects of dietary CrPro on the main physiological signs and production performance of heat-stressed dairy cows

The study indicated that supplementation of chromium trivalent chelates in diets may have an effect on VFAs in rumen of ruminants. Besong et al. (42) found that that dietary supplementation with chromium picolinate had no significant effect on the concentrations of total VFAs, including acetate, propionate, butyrate, valerate, isobutyrate, and isovalerate, in the rumen of dairy cows. Jin and Zhou (47) found that supplementation with Cr-Met had no significant effect on the concentration of each VFA in lambs.

VFAs are also important indicators of overall rumen health and microbial function, and play an important role in the synthesis of milk components such as fat and protein (48–50). Studies have shown that changes in these VFA levels can significantly affect milk production and composition, overall energy status, and even reproductive efficiency of dairy cows (23). Heat stress in dairy cows can cause a variety of physiological changes, among which changes in rumen fermentation process can further affect the production of VFAs (51). Heat stress has been shown to cause a decrease in the ratio of acetic acid to propionic acid produced by rumen fermentation (52). Acetate is one of the important raw materials for milk fat synthesis, and maintaining the normal level of rumen acetic acid is very important to ensure the quality of milk (53). A reduction in acetic acid levels leads to a reduction in milk fat content, which affects the profitability of dairy production (54).

Under heat stress, cows often reduce feed intake, resulting in a decrease in fermentable carbohydrates in the rumen. This may limit the production of propionic acid, one of the raw materials necessary for glucose synthesis (38). Reduced glucose availability can lead to an energy imbalance that affects milk production and quality. Heat stress also causes a decrease in rumen butyric acid levels (55). Butyric acid has a variety of functions, including providing energy to the intestinal epithelium and promoting the immune response. A decrease in butyric acid may exacerbate the negative effects of heat stress on gut health and immune function. The concentrations of valerate, isovalerate, and isobutyric acid in the rumen are very low and have little effect on rumen function, but any changes in rumen fermentation due to heat stress can affect their concentration and subsequent effects, such as contributions to milk fat synthesis. The results of this experiment showed that, consistent with the results of Baumgard et al. (55) the concentration of all VFAs in the control group had a decreasing trend (*p* > 0.05), indicating that heat stress had an adverse effect on rumen fermentation process of dairy cows and reduced the production of all VFAs. The concentration of propionic acid in all groups had a decreasing trend (*p* > 0.05), which may be related to the change of rumen fermentation mode under heat stress. Compared with the control group, rumen VFA in CrL and CrH groups had no significant downward trend (*p* > 0.05), and all VFA in CrL group except propionic acid had an upward trend (*p* > 0.05), indicating that supplementation of 4 and 8 mg/(d·head) CrPro in basic diet could reduce the negative effects of heat stress on rumen fermentation process of dairy cows.

The diversity of the rumen microbiota is crucial for the health and efficient functioning of dairy cows, ensuring metabolic diversity (56). It provides greater stability to resist environmental disturbances and increases the difficulty of colonization by pathogenic microorganisms, thereby enhancing rumen fermentation efficiency (57). The 16S rRNA gene is a highly conserved component of bacterial genomes and serves as an ideal molecular marker for bacterial species identification and classification (58). Rarefaction curves and abundance rank curves of rumen bacteria reflect the evenness of microbial community composition and the reliability of subsequent analyses (59). In alpha diversity analysis, the Chao1 and Observed species indices represent community richness, with higher values indicating a greater number of species. The Shannon and Simpson indices represent species diversity, with higher values indicating greater species diversity in the sample (60). In this study, all curves tended to plateau, indicating that the sequencing depth was sufficient to cover the species in the samples. With increasing CrPro supplementation, there was a trend towards higher Chao1 and Observed species indices (*p* > 0.05), while differences in Shannon and Simpson indices among groups were not significant (*p* > 0.05). This suggests that supplementing the basal diet of lactating cows with 4 and 8 mg/(d·cow) of CrPro may increase rumen microbial richness without altering the existing balance (evenness and dominance) of the rumen microbiota in heat-stressed cows, which is beneficial for enhancing rumen function. However, further studies or larger sample sizes are needed to confirm these findings.

The rumen is a highly complex microbial ecosystem where various microorganisms degrade feed into absorbable nutrients through symbiosis. Results from this study show that at the phylum level, the rumen microbiota of all groups were predominantly composed of *Bacteroidota* and *Firmicutes*, with *Bacteroidota* being the most abundant. As the dose of CrPro increased, the ratio of *Firmicutes* to *Bacteroidota* showed a decreasing trend (*p* > 0.05), indicating that CrPro may influence the structure of the rumen microbiota in dairy cows. *Bacteroidota* mainly consists of cellulose-degrading bacteria responsible for the breakdown of complex plant fibers, leading to increased production of VFAs, especially acetate. Results from this study show that compared with the control group, the abundance of *Bacteroidota* in the CrL group had an increasing trend (*p* > 0.05), which is consistent with the trend of acetate changes. Studies have shown that an increase in the level of Firmicutes is usually associated with increased propionate production. Results from this study indicate that compared with the control group, the abundance of Firmicutes in the CrL and CrH groups had a decreasing trend (*p* > 0.05), which is consistent with the trend of propionate changes. High levels of *Proteobacteria* are often associated with protein-rich diets but may also indicate an imbalance in the rumen environment and increased ammonia production (61). In this study, the CrH group showed an increasing trend in the abundance of *Proteobacteria*, but no significant abnormalities were observed in nitrogen metabolism blood indicators or milk yield indicators. This may be related to the positive effects of CrPro in metabolic pathways related to energy and protein utilization, which offset the negative effects caused by the increased abundance of *Proteobacteria* (61).

*Prevotella*, *Succiniclasticum*, *YRC22*, *Ruminococcus*, *Butyrivibrio*, *Oscillospira*, and *Selenomonas* each constituted >1% of the rumen microbiota across all groups, with *Prevotella*, *Succiniclasticum*, and *YRC22* being the most abundant. *Prevotella*, *YRC22*, *Butyrivibrio*, and *Selenomonas* belong to the phylum *Bacteroidota*, whereas *Succiniclasticum*, *Ruminococcus*, and *Oscillospira* belong to *Firmicutes*. There was no clear trend in the abundance of these genera across groups. However, considering the results of VFA measurements and phylum-level classification, the functional performance of the rumen microbiota improved in all groups. This may be related to functional redundancy in rumen metabolism, where different genera within the same phylum can perform similar metabolic functions (62, 63), but the specific mechanisms need further exploration to verify.

## 5 Conclusion

In summary, our study indicates that dietary supplementation of lactating dairy cows with 8 mg/(d·cow) effectively mitigates heat-stress-induced physiological disturbances, enhances production performance, and alleviates adverse effects on ruminal fermentation without compromising safety. These findings provide robust evidence for selecting an optimal CrPro dose to reduce economic losses attributable to heat stress in dairy operations.

## Data Availability

The original contributions presented in the study are publicly available. This data can be found here: NCBI BioProject, accession PRJNA1308313.

## Glossary

CrPro: Chromium propionate
THI: Temperature-humidity index
BGHI: The black globe-humidity index
WBGT: Wet-bulb globe temperature
RT: Rectal temperature
RR: Respiratory rate
NEFA: Nonestesterified fatty acid
RNA: Ribonucleic acid
AMPK: Adenosine-monophosphate-activated protein kinase
mRNA: Messenger RNA
DMI: Dry matter intake
Cr-Met: Chromium methionate
IBR: Infectious bovine rhinotracheitis
PI-3: Parainfluenza-3
MDA: Malondialdehyde
GSH-Px: Glutathione peroxidase
SOD: Superoxide dismutase
ALP: Alkaline phosphatase
AST: Aspartate aminotransferase
ALT: Alanine aminotransferase
GLU: Glucose
TCHO: Total cholesterol
VFA: Volatile fatty acids
GTF: Glucose tolerance factor
TAG: Triglyceride
HDL: High density lipoprotein cholesterol
LDL: Low density lipoprotein
T-CHO: Total cholesterol
HDL: High density lipoprotein
LDL: Low density lipoprotein
TP: Total protein
ALB: Albumin
Ca^2+^: Serum calcium
Na^+^: Serum natrium
K^+^: Serum kalium
